# Incorporating Environmental Health into Pediatric Medical and Nursing Education

**DOI:** 10.1289/ehp.7166

**Published:** 2004-09-23

**Authors:** Leyla Erk McCurdy, James Roberts, Bonnie Rogers, Rebecca Love, Ruth Etzel, Jerome Paulson, Nsedu Obot Witherspoon, Allen Dearry

**Affiliations:** ^1^National Environmental Education and Training Foundation, Washington, DC, USA; ^2^Medical University of South Carolina, Charleston, South Carolina, USA; ^3^University of North Carolina School of Public Health, Chapel Hill, North Carolina, USA; ^4^George Washington University School of Public Health and Health Services, Washington, DC, USA; ^5^Children’s Environmental Health Network, Washington, DC, USA; ^6^National Institute of Environmental Health Sciences, Research Triangle Park, North Carolina, USA

**Keywords:** education, environmental health, medical student, nursing, nursing student, pediatrics, resident

## Abstract

Pediatric medical and nursing education currently lacks the environmental health content necessary to appropriately prepare pediatric health care professionals to prevent, recognize, manage, and treat environmental-exposure–related disease. Leading health institutions have recognized the need for improvements in health professionals’ environmental health education. Parents are seeking answers about the impact of environmental toxicants on their children. Given the biologic, psychological, and social differences between children and adults, there is a need for environmental health education specific to children. The National Environmental Education and Training Foundation, in partnership with the Children’s Environmental Health Network, created two working groups, one with expertise in medical education and one with expertise in nursing education. The working groups reviewed the transition from undergraduate student to professional to assess where in those processes pediatric environmental health could be emphasized. The medical education working group recommended increasing education about children’s environmental health in the medical school curricula, in residency training, and in continuing medical education. The group also recommended the expansion of fellowship training in children’s environmental health. Similarly, the nursing working group recommended increasing children’s environmental health content at the undergraduate, graduate, and continuing nursing education levels. Working groups also identified the key medical and nursing organizations that would be important in leveraging these changes. A concerted effort to prioritize pediatric environmental health by governmental organizations and foundations is essential in providing the resources and expertise to set policy and provide the tools for teaching pediatric environmental health to health care providers.

With the widespread presence of environmental health hazards in our communities, concern about health risks for children has increased among the general public and the media, as well as among public and private organizations. In a national survey of parents, 41% percent stated they “worry a lot” about their children’s exposure to environmental poisons (Stickler and Simmons 1995). In 1998, the U.S. Environmental Protection Agency (EPA) and the National Institute of Environmental Health Sciences established eight Centers for Children’s Environmental Health and Disease Prevention Research, with four more added in 2000. The U.S. EPA and the Agency for Toxic Substances and Disease Registry (ATSDR) fund 11 Pediatric Environmental Health Specialty Units (PEHSUs). The U.S. EPA and ATSDR funded the first three PEHSUs in 1999. Over the past 10–15 years, the number of children’s environmental health advocacy organizations and federal and state commissions and boards on children’s environmental health has increased. The U.S. EPA created an Office of Children’s Health Protection, and in 1997 President Bill Clinton created the President’s Task Force on Children’s Environmental Health and Safety. Furthermore, total costs of environmentally attributable pollutant-related diseases such as lead poisoning, asthma, and cancer in American children is estimated at $54.9 billion annually (Landrigan et al. 2002).

Despite this increased interest and the economic burden, pediatric medical and nursing education currently lacks the environmental health content necessary to appropriately prepare pediatric health care professionals to recognize, manage, and prevent environmental-exposure–related diseases. Although nurses are the largest group of health professionals and the first—and often only—contact with the health care system for many individuals, most nurses have received no formal training in occupational or environmental health, and registered nurses do not feel well prepared to address environmental health issues in their practice, which has not changed much over time (Pope et al. 1995; Rogers 1991, 1994; Van Dongen 2002). Not all medical schools have faculty equipped to provide this training, and in the 75% of schools that require environmental medicine content, there is only an average of 7 hr environmental medicine instruction (Schenk et al. 1996). More than half of surveyed practicing pediatricians have seen a patient with environmental-exposure–related health issues; however, less than one-fifth are trained in taking an environmental history (Kilpatrick et al. 2002). In a separate survey, only 12% stated they gave advice often on environmental poisons (Stickler and Simmons 1995). Fewer than half of U.S. pediatric residency programs routinely include pediatric environmental health issues in their curricula, other than lead poisoning and environmental exacerbation of asthma (Roberts and Gitterman 2003).

Leading health institutions have called for improvements in health professionals’ environmental health education. The Institute of Medicine (IOM) recommends the integration of environmental health concepts into all levels of medical and nursing education and has issued competencies that graduating medical and nursing students should demonstrate, including the ability to take an environmental exposure history, to understand the influence of environmental exposures on human health, and to communicate environmental risks and prevention strategies (IOM 1988, 1991; Pope and Rall 1995; Pope et al. 1995). Several medical and nursing organizations have expressed their support of environmental health education for health care professionals by endorsing the Health Professionals and Environmental Health Education Position Statement [National Environmental Education and Training Foundation (NEETF) 2004; Rogers 2004].

Training of health care providers on the special environmental health issues related to children is almost nonexistent, yet such education is necessary given the biologic, psychological, and social differences between children and adults. To address this need, two working groups of pediatric physicians and nurses conducted an assessment of and identified points of insertion in the current medical and nursing education structures where environmental health content could be incorporated.

## Materials and Methods

Because there are so many differences between medical and nursing education, separate working groups with distinct expertise in the respective fields were created to assess each of those areas. Members of the medical and nursing working groups were selected because of their expertise and knowledge about *a*) environmental health education or medical or nursing education in general, *b*) accreditation programs and systems, or *c*) curriculum development.

These working groups met by phone and shared materials electronically. Their task was to describe the two educational processes from undergraduate education to continuing education and to identify potential leverage points that could be used to increase the amount of children’s environmental health information in the education of physicians and nurses. All working group members were involved in all the discussions about all the potential leverage points, irrespective of whether the particular point involved education, accreditation, or curriculum development.

## Results and Discussion

In this section we first outline the medical and nursing educational processes, including coursework, clinical practice experiences, and formal examinations. Tables 1 and 2 show medical and nursing education accrediting and licensing organizations. Tables 3 and 4 present the medical and nursing educational processes and leverage points for insertion of pediatric environmental health. We use these outlines to demonstrate how pediatric environmental health content could be most strategically inserted into existing curricula. This is envisioned as a multipronged, ongoing process because no single change will bring about the desired outcome of increased content. The following recommendations should be used to introduce pediatric environmental health education to achieve the standards outlined in the IOM recommendations.

### Medical education.

Medical school is the first phase in the education structure of physicians. The curriculum provides instruction in the underlying sciences of medical practice (basic sciences) and in information-gathering, decision-making, and patient-management skills. Students take, and in some schools must pass, step 1 and step 2 of the U.S. Medical Licensing Examination (USMLE), between the second and third years and before graduation from medical school, respectively. Upon successful completion of medical school, students receive their MD degree and undertake the next phase of medical education, residency.

Residency offers physicians an organized educational program with guidance and supervision of the resident to facilitate the resident’s professional and personal development while ensuring safe and appropriate care for patients. Residents take step 3 of the USMLE during or after their first year of residency. A physician interested in a career in pediatric environmental health could choose a residency in one of three specialties: family medicine, pediatrics, or preventive medicine. Residents are prepared to undertake independent medical practice upon satisfactory completion of a residency and can apply to take a certifying examination to certify competency in their specialty.

Fellowships provide an optional, usually 3-year-long period of training after the completion of residency, for subspecialization. Fellowships offer a path to a faculty position in a medical school and/or a residency training program. When a specialty board offers certification in the field, completion of a fellowship can be a step toward certification by a specialty board or subboard. There is no specialty board or specialty certification available in pediatric environmental health. Currently, the Ambulatory Pediatric Association (APA) offers a 3-year pediatric environmental health fellowship training program, and the Cincinnati Children’s Hospital offers the general pediatric research fellowship with a strong emphasis on pediatric environmental health.

In most states, physicians must obtain 150 continuing medical education (CME) credits every three years on any topic they choose to maintain a medical license. Credits can be obtained by attending seminars, lectures, or workshops; by pursuing web-based programs; or by doing research projects, writing manuscripts, or reading material and taking a test.

Recertification ensures that physician specialists remain up-to-date in their specialty by requiring them to pass an exam to remain certified. Physicians certified by the American Board of Pediatrics after 1987 must be recertified every seven years, and those certified by the American Board of Preventive Medicine after 1997 must be recertified every 10 years. Currently, there is no requirement to include children’s environmental health content in the recertifying examinations for pediatrics or preventive medicine.

### Nursing education.

To become a nurse, individuals must accomplish one of three undergraduate programs of study: a 2-year associate degree (AA), a 3-year diploma, or a 4-year baccalaureate degree (BSN). The curricula for the AA and diploma programs provide students instruction in the underlying sciences of nursing practice as well as the application of information in clinical settings. Nurses from AA and diploma programs can continue undergraduate nursing education with completion of the BSN. The first 2 years of the baccalaureate curriculum focus on the underlying sciences for practice, and the last 2 years provide opportunities for developing information-gathering, decision-making, and patient-care skills in a broad array of clinical and public health settings, including hospitals, clinics, public health departments, government, and workplace settings.

At the completion of the basic nursing program (AA, diploma, or BSN), graduates must pass the National Council Licensure Examination (NCLEX) to obtain a Registered Nurse (RN) license.

Baccalaureate-educated nurses can go to graduate school to develop advanced knowledge in a particular nursing specialty. Depending on the specialty and outcome focus of the nursing program of study, graduate nursing education curriculum may include advanced basic sciences, evidence-based nursing practice, health policy, advanced nursing assessment, research coursework, clinical coursework, and program planning and evaluation, usually with supervised practicum experiences in the specialty—for example, pediatrics, adult health, and occupational and environmental health.

Upon completion of the master’s degree, nursing graduates of certain clinically focused specialties must become certified by their state board of nursing based on graduation from an approved program and/or completion of the national board exam. National board exams are available for all nurse practitioner specialties, certified nurse midwives, certified nurse anesthetists, and some clinical nurse specialists. There have been significant efforts by nursing leaders to make the board exam requirements consistent across specialties.

Nurses with a master’s degree may continue for doctoral education in a specialty of their choosing. These specialties may or may not be in nursing (e.g., nursing, public health, educational psychology, anthropology, physiology, or other fields may be selected). The doctoral degree generally provides the graduate with advanced knowledge for teaching or research in nursing. There are no further board examinations or certifications for doctoral program graduates.

Nurses may also become certified in their chosen specialty through national nursing specialty certification boards, which generally require examination, experience, and continuing/academic education. National specialty boards offer programs to maintain/renew certification every 5–7 years. Requirements generally include continuing education contact hours at approved courses, self-assessment exercises, practice requirements, and/or retaking the national board examinations. Continuing education course approval is given by specialty organizations and must meet nationally recognized quality standards.

### Potential leverage points in medical education.

Table 3 presents medical education and the corresponding points of insertion, strategies, and influential groups for incorporating children’s environmental health.

#### Medical school.

The working group identified several organizations and strategies that could be used to insert children’s environmental health content into medical schools. These include medical students and medical-student organizations, the creation of designated faculty leaders within schools of medicine, the Association of American Medical Colleges (AAMC), the Liaison Committee on Medical Education (LCME), and the National Board of Medical Examiners (NBME).

Medical students and medical-student organizations have some influence over the curriculum of their own medical school (Atkins et al. 1998; Grayson et al. 1999; Rollins et al. 1999). Students can promote the addition of children’s environmental health curricular elements and case studies at their medical schools through course evaluations and representation on curricular committees (Steyer et al. 2003). Students may also introduce topics through noncredit courses and activities, such as with the modules and tool kits on various topics provided by the American Medical Student Association or the idea book of projects issued by the American Medical Association (AMA) student section. Educating medical students on environmental health will prepare them to promote this issue at their medical schools.

Medical school faculty members are key to implementing curricula and influencing career choices of students by setting examples and providing direct counseling (Goldman et al. 1999; Schwartz et al. 1995). There should be a concentrated effort to develop qualified faculty members at pediatric training programs. The role of a faculty leader in pediatric environmental health would be to coordinate all children’s environmental health activities at the school, teach appropriate curricular material, provide case material that could be used in courses taught by others, and coordinate with colleagues around the country. Primary-care residency faculty trained in environmental/occupational health can increase the environmental/occupational health education offered at their schools (Frazier et al. 1999). Faculty leaders that have an interest in teaching environmental health are essential to integrating environmental health content into the curriculum and provide the impetus for change throughout a program. In several studies about how to integrate prevention-related topics, a key determinant of success was shown to be faculty and institutional leadership (Lindberg 1998; Sachdeva 2000; Skochelak et al. 2001; Susman and Pascoe 2001).

The AAMC, LCME, and NBME have no direct input into medical school curricula. The AAMC, however, sponsors regional meetings where innovative curricular activities are presented to others for consideration. Similar presentations are also made at APA meetings. It is recommended that faculty members teaching children’s environmental health or using children’s environmental health case material submit abstracts to present their activities at these meetings.

#### Residency training.

At the residency training level, leverage points include directors of residency education, pediatric department chairs, chief residents, residency review committees, and pediatric primary care education guidelines. Chief residents have influence within each residency-training program over the scheduling of conferences and educational activities and could require all residents to complete education on children’s environmental health. The American Academy of Pediatrics (AAP), with funding from the U.S. EPA, has created a 1-day program to train pediatric chief residents about children’s environmental health.

Pediatric residency review committees (RRC) can play a role in increasing children’s environmental health by requiring pediatric residency education to include children’s environmental health content. For programs that do not have faculty members qualified to teach that content, a web-based self-instructional module could be developed for residents, similar to the ATSDR’s web-based continuing education case studies. The ATSDR and U.S. EPA, through their cooperative agreement with the Association of Occupational and Environmental Clinics (AOEC), could task a PEHSU with developing and disseminating such a module. Training programs regarding the content and use of the module could be developed for residency program directors and chief residents.

Another strategy is to include teaching about children’s environmental health in the guidelines for pediatric primary care education. In one academic setting, the incorporation of a pediatric environmental health course resulted in physicians’ increased consideration of environmental causes for illness (Bearer and Phillips 1993). The APA has developed a set of guidelines for pediatric primary care education at the residency level and could include children’s environmental health in future iterations, possibly drawing from the competencies that the APA developed for specialists in pediatric environmental health, described below.

#### Fellowships and specialty certification.

Another strategy to prepare more experts in the field, who could meet growing patient demand and train the next generation of pediatricians, is to increase the number of fellows in pediatric environmental health. The Centers for Children’s Environmental Health and Disease Prevention Research and the PEHSUs could provide the platforms upon which to build these fellowship programs. In 2003, the APA published a list of competencies for specialists in pediatric environmental health (Etzel et al. 2003). Twenty-seven competencies, each accompanied by a list of suggested performance indications, were developed under three separate perspectives: academic, individual patient care, and community advocacy. These competencies are intended to help structure the training experience, achieve consensus with respect to expectations of fellows and faculty, provide opportunities for fellows to assess their own needs or gaps in training, and identify the expertise of fellowship graduates to potential employers.

The creation of a specialty board offering certification in pediatric environmental health serves as a leverage point to formalize pediatric environmental health as a subspecialty and allow physicians to specialize and become leaders in the field. The American Board of Preventive Medicine (ABPM) offers specialty certification in occupational medicine or general preventive medicine, and the American Board of Emergency Medicine, the American Board of Pediatrics (ABP), and the ABPM have a subboard in medical toxicology. Some physicians choose one of these specialties as a route to a career in pediatric environmental health. The ABP and/or the ABPM could seek permission from the American Board of Medical Specialties (ABMS) to develop a sub-board in pediatric environmental health.

#### Continuing medical education.

CME is a means to provide environmental health education to physicians postresidency and at later stages in their careers. CME on environmental health issues, such as environmental asthma triggers, has been effective in improving the health of patients and decreasing associated medical costs (Clark et al. 2000). The PEHSUs currently provide some CME activities; however, this working group recommends increasing the opportunities for practicing physicians, nurse practitioners, and other child health care providers to learn about children’s environmental health. Professional organizations, such as the AAP, the APA, the American Public Health Association (APHA), and other nongovernmental organizations, could provide children’s environmental health CME programming.

#### Pediatric practice.

Medical insurance companies influence medical practice. If services are reimbursable, they are more likely to be offered to patients. Fellowships can be funded in part from service-related income. The organizations involved in children’s environmental health, such as the AAP and the AOEC, should request reimbursement from insurance companies for environmentally related health care services provided to children and should lobby state legislatures and state insurance boards for such coverage.

### Potential leverage points in nursing education.

Table 4 shows the steps in nursing education and corresponding points of insertion, strategies, and influential groups for inserting children’s environmental health.

#### Undergraduate nursing education.

Many undergraduate nursing education organizations and groups could introduce children’s environmental health content into nursing curricula: nursing students and nursing-student organizations, the National League for Nursing Accrediting Commission (NLNAC), and the Commission on Collegiate Nursing Education (CCNE).

Nursing students have influence over the content of the curriculum in their schools. Nursing-student organizations, by linking students in various schools and providing information at meetings for students to take back to their schools, can influence the curricular content. Nursing professionals and environmental organizations interested in children’s environmental health should strive for input into local, regional, and national nursing student groups to teach them about the importance of this topic. One existing effort in this realm is the AOEC’s sponsorship of two focus sessions on occupational and environmental health nursing at the National Student Nurses Association annual meeting.

NLNAC and CCNE should work to include examples of children’s environmental health issues throughout the undergraduate nursing curriculum. For example, cases related to children’s environmental health could be used for curriculum content on epidemiology that all nursing students take. Case studies covering a number of issues are available through the AOEC, the Great Lakes Center for Occupational and Environmental Safety and Health, and the ATSDR.

When nursing students do their fieldwork, they should be encouraged to work with local and regional agencies that focus on children’s environmental health issues.

AA, diploma, and baccalaureate degree nurses must pass the NCLEX to be licensed to practice as an RN. Influencing the NCLEX’s content is difficult because content is determined by a survey of the work of practicing nurses, and most practicing nurses have limited environmentally related activities in their day-to-day nursing activities. However, as practice changes to embrace environmental health content, the inclusion of environmental-health–related questions will influence curricula.

As in medical education, nursing faculty with pediatric environmental health background can significantly influence curricular content and practice activities at the undergraduate, graduate, and continuing nursing education levels. Development of children’s environmental health nursing faculty leaders is essential.

#### Graduate nursing education and certification.

The National Organization of Nurse Practitioner Faculties (NONPF) and the Association of Faculties of Pediatric Nurse Practitioner Programs should use their influence on the curricula and standards for education and competencies to increase children’s environmental health content in the programs for advanced practice nurses. For example, a competency requirement on environmental health education has been accepted by NONPF, which could expand this activity and ensure its inclusion in the curriculum of all nurse practitioner programs.

Organizations such as the Pediatric Nursing Certification Board (PNCB); the American Nurses Credentialing Center; the National Certification Corporation for Obstetrical, Gynecological, and Neonatal Nurses; the American College of Nurse Midwives; and the American Board of Occupational Health Nurses, which develop the certifying examinations for their respective specialties, should include children’s environmental health material in their examinations. Environmental health was included in a recent PNCB self-assessment exercise for certification maintenance of pediatric nurse practitioners.

#### Continuing nursing education.

As with CME, continuing education for nurses is a key point at which to educate nurses about pediatric environmental health. One such example is the innovative children’s environmental health continuing education program conceived by the University of Maryland School of Nursing Environmental Health Education Center in conjunction with the American Nurses Association. It should be expanded to include other topics and repeated on a regular basis to provide education to newly trained nurses or nurses with a newly identified interest in children’s health and the environment. Organizations such as APHA, the National Association of Pediatric Nurse Practitioners, and the School Nurses Association, should sponsor pre- or postconference workshops on children’s health and the environment for nurses at their annual conferences. The train-the-trainer format would be a useful technique for spreading children’s environmental health expertise.

## Conclusion

We have identified several strategic opportunities to incorporate much-needed pediatric environmental health into the existing medical and nursing education process. Medical, nursing, and public health organizations, as well as patients, have expressed the need for health care providers to be better equipped to recognize and treat environmentally caused illness. We provide this comprehensive list of insertion points to guide medical and nursing education accrediting bodies, licensing bodies, and other key personnel that determine and influence medical and nursing curricula. Future efforts in this area should include evaluation of the existing pediatric environmental health education programs to determine the most effective formats for incorporating this subject. In addition, because knowledge and research in many areas of children’s environmental health are still developing, future efforts should focus on ensuring curriculum development and updates as the field advances. A concerted effort to prioritize pediatric environmental health by governmental organizations and foundations will be essential in providing the resources and expertise to set policy and provide the tools for teaching pediatric environmental health to health care providers.

## Appendix 1: Medical and Nursing Education Working Groups

The following individuals participated in the review of medical education opportunities: Rob Amler, MD, MS—(then) Chief Medical Officer, ATSDR; Lois Colburn—Assistant Vice President for Minority and Community Programs, AAMC (Curriculum Development); Susan Cummins, MD, MPH—(then) Director, Board of Children Youth and Families, National Academy of Sciences, IOM; Deborah Danoff, MD—Assistant Vice President for Medical Education, AAMC; Ruth Etzel, MD, PhD—George Washington University School of Public Health and Health Services; Leyla Erk McCurdy, MPhil—National Environmental and Education Training Foundation, Project Director; Jerome A. Paulson, MD—(then) Soros Fellow, Children’s Environmental Health Network, and Co-director, Mid-Atlantic Center for Children’s Health and the Environment, Region 3 PEHSU; James Roberts, MD, MPH—Assistant Professor, Department of Pediatrics, Medical University of South Carolina; Chris Rosheim, DDS, MPH—(then) Health Education Specialist, ATSDR; Bernhard L. Wiedermann, MD—Associate Professor and Vice Chair for Education, Department of Pediatrics, George Washington University School of Medicine, and Director, Medical Education and Pediatric Residency Training Program, Children’s National Medical Center, Washington, DC.

The following individuals participated in the review of nursing education opportunities: Robert Atkins, MSN, CRNP—Director of Pediatric Nurse Practitioner Program, Temple University, Department of Nursing; Cathie Burns, PhD, RN, CPNP—Professor Emeritus, School of Nursing, Oregon Health Sciences University; Hurdis Griffith, PhD, RN—Dean of Rutgers School of Nursing; Barbara Kelley, EdD, MPH, MS—Associate Professor and Director for Graduate Nursing Program Northeastern University; Rita Lourie, MSN, RN—Director of Academic and Community Outreach, Temple University, Department of Nursing; Leyla Erk McCurdy, MPhil—National Environmental and Education Training Foundation; Grace K. Paranzino, MS, RN, CHES, FAAOHN—Assistant Professor, Drexel University School of Medicine, Department of Family, Community and Preventive Medicine, and Adjunct Assistant Professor, Department of Environmental and Occupational Health, Drexel University School of Public Health; Dorothy Powell, EdD, RN, FAAN—Associate Dean, College of Pharmacy, Nursing, and Allied Health Sciences, Howard University/Director Mississippi Delta Project; Bonnie Rogers, DrPH, COHN-S, FAAN—Director of Occupational/Health Safety and Nursing Programs, University of North Carolina School of Public Health; Izzat Sbeih, MPH—Health Policy Analyst, American Public Health Association.

## Figures and Tables

**Table 1:** Organizations involved with medical accreditation and licensing.

Organization	Function	Consisting of representatives from	Subsidiary organizations
Liaison Committee on Medical Education (LCME)	Review and approval of medical school curricula; accreditation of medical schools	American Medical Association (AMA); Association of American Medical Colleges (AAMC)	
National Board of Medical Examiners (NBME)	Development of U.S. medical licensing examination		
Accreditation Council for Graduate Medical Education (ACGME)	Development of methods to evaluate and promote the quality of graduate medical education; accreditation of programs in graduate medical education according to established standards	AAMC; AMA; American Board of Medical Specialties (ABMS); American Hospital Association (AHA); Council of Medical Specialty Societies (CMSS)	Residency review committees
Residency review committees (RRC): each specialty has a corresponding RRC	Accreditation review of residency training programs; review and revision of specialty requirements	Corresponding specialty board (American Board of Pediatrics and American Academy of Pediatrics for the pediatric RRC); AMA Council on Medical Education	
American Board of Medical Specialties (ABMS)	Assist specialty boards to promote the quality and efficiency of the process of evaluating and certifying physician specialists; act as spokesperson for specialty boards		Specialty boards
Specialty boards	Provide comprehensive exams; certify those who have satisfied requirements		
Accreditation Council for Continuing Medical Education (ACCME)	Promote and develop principles, policies, and standards for CME and apply them to the accreditation of institutions and organizations offering CME	ABMS; AHA; AMA; AAMC; CMSS; Association for Hospital Medical Education; Federation of State Medical Boards; nonvoting members: resident physician section of AMA; U.S. Department of Health and Human Services; chair of the residency committee council	

**Table 2:** Organizations involved with academic or legislated programs to assure quality of nursing practice.

Organization	Function	Consisting of representatives from
National League for Nursing Accrediting Commission (NLNAC)	Approve nursing programs of study	Independent body derived from the National League of Nursing
Commission on Collegiate Nursing Education (CCNE)	Approve baccalaureate and graduate nursing curriculum, faculty, administration, and programs	Independent body derived from the American Association of Colleges of Nursing
National Council of State Boards of Nursing (NCSBN)	Administration of National Council Licensure Examination–RN	State boards of nursing
State boards of nursing	Provide licensure for registered nurses and certification for nurse practitioners, certified nurse midwives, and other graduate specialties that must be legally certified to practice in the state	
Specialty boards		
Pediatric Nursing Certification Board (PNCB)	Professional certification of pediatric nurse practitioners and nurse specialists	American Academy of Pediatrics; Association of Faculties of Pediatric Nurse Practitioners; National Association of Pediatric Nurse Practitioners; Society of Pediatric Nurses
American Nurses Credentialing Center (ANCC)	Professional certification of pediatrics, adult, family, and geriatrics nurse practitioners	
American Academy of Nurse Practitioners (AANP)	Professional certification of adult and family nurse practitioners	
National Certification Corporation for Obstetrical, Gynecological, and Neonatal Nurses (NCC)	Professional certification of women’s health care nurse practitioners	
American College of Nurse-Midwives (ACNM)	Professional certification of certified nurse midwives	
Nurse practitioner faculty organizations		
National Organization of Nurse Practitioner Faculties (NONPF)	Influence on curricula and standards for education/competencies for nurse practitioner programs and their graduates	Faculty from all nurse practitioner specialties
Association of Faculties of Pediatric Nurse Practitioners (AFPNP)	Influence on curricula and standards for education/ competencies for pediatric nurse practitioner programs and their graduates	Faculty from pediatric nurse practitioner programs

**Table 3:** Medical education structure and leverage points for insertion of pediatric environmental health.

Medical education	Leverage points
Medical school (MD curriculum)	Association of American Medical Colleges, faculty, National Board of Medical Examiners (NBME), Liaison Committee on Medical Education, students, student organizations
Residency	Accreditation Council for Graduate Medical Education (ACGME), American Board of Medical Specialties (ABMS), chief residents, directors of residency education, NBME, pediatric department chairs, primary care pediatric education guidelines, residency review committees, specialty boards
Fellowship (optional)	ABMS, fellowships, specialty boards
Continuing medical education (CME)	Accreditation Council for Continuing Medical Education (ACCME) professional organizations that provide CME
Recertification	Specialty boards

**Table 4:** Nursing education structure and leverage points for insertion of pediatric environmental health.

Nursing education	Leverage points
Undergraduate nursing education	National League for Nursing Accrediting Commission, Commission on Collegiate Nursing Education, students, student organizations, National Student Nurse Association, nursing professionals and faculty, fieldwork, specialty organizations
Associate degree, diploma, baccalaureate degree	
Graduate nursing education	Pediatric Nursing Certification Board; American Nurses Credentialing Center; National Certification Corporation for Obstetrical, Gynecological, and Neonatal Nurses; National Organization of Nurse Practitioner Faculties; Association of Faculties of Pediatric Nurse Practitioner Programs; specialty organizations
Master’s degree (nurse specialists, nurse practitioner, certified nurse midwife, certified nurse anesthetist)	
Doctoral degree	Specialty organizations
Continuing education	Specialty organizations, workshops at conferences

## References

[b1-ehp0112-001755] Atkins KM, Roberts AE, Cochran N (1998). How medical students can bring about curricular change. Acad Med.

[b2-ehp0112-001755] Bearer CF, Phillips R (1993). Pediatric environmental health training. Impact on pediatric residents. Am J Dis Child.

[b3-ehp0112-001755] Clark NM, Gong M, Schork MA, Kaciroti N, Evans D, Roloff D (2000). Long-term effects of asthma education for physicians on patient satisfaction and use of health services. Eur Respir J.

[b4-ehp0112-001755] Etzel RA, Crain EF, Gitterman BA, Oberg C, Scheidt P, Landrigan PJ (2003). Pediatric environmental health competencies for specialists. Ambul Pediatr.

[b5-ehp0112-001755] Frazier LM, Berberich NJ, Moser R, Cromer JW, Hitchcock MA, Monteiro FM (1999). Developing occupational and environmental medicine curricula for primary care residents: project EPOCH-Envi. Educating Physicians in Occupational Health and the Environment. J Occup Environ Med.

[b6-ehp0112-001755] Goldman RH, Rosenwasser S, Armstrong E (1999). Incorporating an environmental/occupational medicine theme into the medical school curriculum. J Occup Environ Med.

[b7-ehp0112-001755] Grayson MS, Newton DA, Klein M, Irons T (1999). Promoting institutional change to encourage primary care: experience at New York Medical College and East Carolina University of Medicine. Acad Med.

[b8-ehp0112-001755] IOM (Institute of Medicine) 1988. Role of the Primary Care Physician in Occupational and Environmental Medicine. Washington, DC:National Academy Press. 25077202

[b9-ehp0112-001755] IOM (Institute of Medicine) 1991. Addressing the Physician Shortage in Occupational and Environmental Medicine. Washington, DC:National Academy Press. 25077200

[b10-ehp0112-001755] Kilpatrick N, Frumkin H, Trowbridge J, Escoffery C, Geller R, Rubin I (2002). The environmental history in pediatric practice: a study of pediatricians’ attitudes, beliefs, and practices. Environ Health Perspect.

[b11-ehp0112-001755] Landrigan PJ, Schechter CB, Lipton JM, Fahs MC, Schwartz J (2002). Environmental pollutants and disease in American children: estimates of morbidity, mortality, and costs for lead poisoning, asthma, cancer, and developmental disabilities. Environ Health Perspect.

[b12-ehp0112-001755] Lindberg MA (1998). The process of change: stories of the journey. Acad Med.

[b13-ehp0112-001755] NEETF 2004. Health Professionals and Environmental Health Education Position Statement. Washington, DC:National Environmental Education and Training Foundation. Available: http://www.neetf.org/Health/PositionStatement2.pdf [accessed 25 October 2004].

[b14-ehp0112-001755] Pope AM Rall DP eds, for Committee on Curriculum Development in Environmental Medicine, Institute of Medicine. 1995. Environmental Medicine: Integrating a Missing Element into Medical Education. Washington, DC:National Academy Press. 25121193

[b15-ehp0112-001755] Pope AM Snyder MA Mood LH eds, for Committee on Enhancing Environmental Health Content in Practice, Institute of Medicine. 1995. Nursing, Health, and the Environment: Strengthening the Relationship to Improve the Public’s Health. Washington, DC:National Academy Press. 25121198

[b16-ehp0112-001755] Roberts JR, Gitterman BA (2003). Pediatric environmental health education: a survey of US pediatric residency programs. Ambul Pediatr.

[b17-ehp0112-001755] Rogers B (1991). Occupational health nursing education: content in baccalaureate programs. AAOHN J.

[b18-ehp0112-001755] Rogers B (1994). Linkages in environmental and occupational health: assessing, detecting, and containing exposure sources. AAOHN J.

[b19-ehp0112-001755] Rogers B (2004). Environmental health hazards and health care professional education. AAOHN J.

[b20-ehp0112-001755] Rollins LK, Lynch DC, Owen JA, Shipengrover JA (1999). Moving from policy to practice in curriculum change at the University of Virginia School of Medicine, East Carolina University School of Medicine, and SUNY-Buffalo School of Medicine. Acad Med.

[b21-ehp0112-001755] Sachdeva AK (2000). Faculty development and support needed to integrate the learning of prevention in the curricular of medical schools. Acad Med.

[b22-ehp0112-001755] Schenk M, Popp SM, Neale AV, Demers RY (1996). Environmental medicine content in medical school curricula. Acad Med.

[b23-ehp0112-001755] Schwartz BS, Pransky G, Lashley D (1995). Recruiting the occupational and environmental medicine physicians of the future: results of a survey of current residents. J Occup Environ Med.

[b24-ehp0112-001755] Skochelak S, Barley G, Fogarty J (2001). What did we learn about leadership in medical education? Effecting institutional change through the Interdisciplinary Generalist Curriculum Project. Acad Med.

[b25-ehp0112-001755] Steyer TE, Ravenell RL, Mainous AG, Blue AV (2003). The role of medical students in curriculum committees. Teach Learn Med.

[b26-ehp0112-001755] Stickler GB, Simmons PS (1995). Pediatricians’ preference for anticipatory guidance topics compared with parental anxieties. Clin Pediatr.

[b27-ehp0112-001755] Susman J, Pascoe J (2001). Recommendations to Institutions. Acad Med.

[b28-ehp0112-001755] Van Dongen CJ (2002). Environmental health and nursing practice: a survey of registered nurses. Appl Nurs Res.

